# Presleep focusing on positive spontaneous thoughts enhanced the possibility of dreaming of them

**DOI:** 10.3389/fpsyg.2022.1042857

**Published:** 2022-12-14

**Authors:** Jiaxi Wang, Bin Song, Xiaoling Feng, Heyong Shen, Ruoqiao Liu

**Affiliations:** ^1^School of Psychology, South China Normal University, Guangzhou, China; ^2^Guangzhou University of Chinese Medicine, Guangzhou, China; ^3^Institute of Analytical Psychology, City University of Macau, Macao, China; ^4^School of Psychology, Shaanxi Normal University, Xi’an, China

**Keywords:** Calvin Hall, continuity hypothesis, current concern, indirect association, metaphor, priming, sleep

## Abstract

**Introduction:**

Dreaming is the subjective experience during sleep. A spontaneous thought is a thought that comes to one’s mind involuntarily. This study investigated whether presleep focusing on a positive spontaneous thought enhanced the possibility of dreaming of the thought.

**Methods:**

Ninety-seven participants were quasi-randomly assigned to an expression condition (focus on an spontaneous thought for 5-Min before sleeping; N = 45) and a control condition (think about anything for 5-Min before sleeping; N = 45). Participants completed a dream diary upon waking. Then, both participants themselves (the selfrating method) and external judges (the external-rating method) rated the correlation between the positive spontaneous thought and the dream.

**Results:**

The result of the external-rating method indicated that presleep focusing on positive spontaneous thoughts enhanced the possibility of dreaming of the thoughts. In addition, the external-rating method found that presleep focusing on positive spontaneous thoughts enhanced the possibility of dreaming of thoughts that were related to the positive spontaneous thoughts but not the positive spontaneous thoughts themselves.

**Discussion:**

These results supported the current concern theory which suggests that one’s current concerns increase responses to cues related to the concerns implicitly. In addition, these results supported the continuity hypothesis which states that dreaming is in continuous with waking life, and thus the intensity of a daily concern may be related to the possibility of dreaming of the daily concern.

## Introduction

Spontaneous thoughts are widely existed in people’s life. Some of them contain negative emotions, while others contain positive emotions. Negative spontaneous thoughts (or intrusive thoughts) can be seen as a mark for many diseases, such as depression (Wenzlaff et al., 1988; Purdon and Clark, 1993; Reynolds and Brewin, 1998), anxiety (Purdon and Clark, 1993), obsessive–compulsive disorder (Salkovskis, 1989; Purdon and Clark, 1993), and post-traumatic stess disorder (Ehlers and Steil, 1995; Reynolds and Brewin, 1998; Ehlers and Clark, 2000). On the other hand, except for negative thoughts, there are also positive spontaneous thoughts. Research has shown that positive spontaneous thoughts are also related to personal psychological health. For example, a positive spontaneous thought related to gym may promote individual willfulness to do physical exercise related to the spontaneous thought (Rice and Fredrickson, 2017). Thus, positive spontaneous thoughts may help people to build healthy physical behavior (Fredrickson, 2013; Fredrickson et al., 2015), reduce problems with chronic illness, and enhance psychological social function (Keyes, 2005). In addition, It is argued that positive spontaneous thoughts may have substantial effects on happiness and health (Rice et al., 2016).

Dreaming is the subjective experience during sleep. As it is argued that waking-life experiences can be incorporated into dreams (Freud, 1900), many research explored potential factors that can influence the possibility of the incorporation of waking-life experiences being incorporated into dreams (e.g., Schredl and Hofmann, 2003; Nielsen et al., 2004; Wamsley and Stickgold, 2009; Malinowski and Horton, 2014; van Rijn et al., 2015; Wang et al., 2020). To our knowledge, most of this kind of evidence was correlational in nature, because there was a difficulty to manipulate dream content directly (for a talk, see Schredl, 2003). Yet still some studies showed a causal relationship for this topic (e.g., Nikles et al., 1998; Wegner et al., 2004; Taylor and Bryant, 2007; Kröner-Borowik et al., 2013; Malinowski et al., 2019; Feng and Wang, 2022; Wang et al., 2022). These studies can be divided into two kinds. One suggested that presleep suppressing a target thought enhanced the possibility of dreaming of the thought (e.g., Wegner et al., 2004; Taylor and Bryant, 2007; Bryant et al., 2011; Kröner-Borowik et al., 2013; Malinowski et al., 2019; Wang et al., 2022), and the other indicated that presleep focusing on a target thought increased the possibility of dreaming of the thought (e.g., Nikles et al., 1998; Wang et al., 2022). Yet evidence for the latter kind of finding was inconsistent, because Wegner et al. (2004) did not find that presleep focusing on a target thought increased the possibility of dreaming of the thought, so there was still a need to repeat the finding. Wang et al. (2022) found that presleep focusing on an intrusive thought enhanced the possibility of dreaming of the thought. As stated above, in addition to negative spontaneous thoughts (intrusive thoughts), there are also positive spontaneous thoughts It has been found that the emotional intensity of a waking-life experience, rather than the emotional valence of a waking-life experience, could affect the possibility of dreaming of the experience (e.g., see Malinowski and Horton, 2014). Hence, presleep focusing on positive spontaneous thoughts may also enhance the possibility of dreaming of the thoughts. Here we explored this idea. We used a similar paradigm with Wang et al. (2022). An expression condition was used to instruct participants to focus on a positive spontaneous thought, and a control condition was used to instruct participants to think about anything they want. We first hypothesized that the expression condition had more possibility of dreaming of the positive spontaneous thought than the control condition.

In addition, there were two ways to identify potential correlation between a spontaneous thought and a dream. One was the self-rating method which instructed participants to give scores to represent correlations between target thoughts and dreams. The other was the external-rating method which instructed external judges to rate correlations between target thoughts and dreams. The self-rating method may be affected much by the individual differences in the ability to identify correlations between target thoughts and dreams, which may, in turn, affect results of comparisons between different conditions. So the external-rating method might be more suitable for group comparisons. The above statement may be supported by the finding concerning the topic of a dream rebound effect. The dream rebound effect referred to an increased possibility for a suppressed thought appearing in dreams (e.g., Wegner et al., 2004). Wegner et al. (2004) used the self-rating method to find the dream rebound effect. Yet in Wang et al.’s (2022) study, the self-rating method did not find the dream rebound effect, whereas the external-rating method did. The inconsistent results between Wegner et al. (2004) and Wang et al. (2022) implied that the external-rating method was more suitable for group comparisons. Yet still, there was a need for more research to explore this idea. So here similar to Wang et al. (2022), we used both the external-rating method and the self-rating method for our purpose to make group comparisons.

Furthermore, it has been arguing that there are metaphorical expressions of waking-life experiences (e.g., see Malinowski and Horton, 2015). The existence of dream metaphors increased the difficulty to rate correlations between waking-life experiences and dreams. These dream metaphors mean that dreams represent waking-life experiences indirectly (e.g., Lakoff, 1993). From the perspective of psychoanalysis, the way to decode dream metaphors was to let a dreamer give associations of each element of his (her) own dream (e.g., see Freud, 1900). If a waking-life experience is related to any association of the dream, then the correlation between the dream and the waking-life experience will be identified. From the perspective of language and memory research, the semantic priming refers to the finding that a target word (e.g., black) is processed more accurately and faster when it is preceded by a related prime word (e.g., white) than when it is preceded by an unrelated prime word (e.g., apple). The priming effect between a directly related prime and target is greater than priming effect between an indirectly related prime and target (e.g., see Kenett et al., 2017). For example, the word ‘mane’ and the word ‘lion’ might be directly connected, whereas the word ‘mane’ and the word ‘tiger’ might be connected only through the mediating word ‘lion’. The above way that psychoanalysist used to decode dream metaphors is similar to the situation that in the linguistic area a prime and a target have an indirect relationship of connection. Here we used a similar way to decode dream metaphors. If any association of a waking-life experience is related to a dream, it can be concluded that the waking-life experience is incorporated into the dream indirectly. In Wang et al.’s (2022) study, there was a stream of consciousness task, in which participants recorded any thoughts that popped into their minds. So recordings of the stream of consciousness task contain a series of words. In the stream of consciousness task, words that were next to target thoughts may be related to the target thoughts. In this study, we viewed these kinds of words as thoughts that were relevant to target thoughts but not target thoughts themselves. If dreams were related to these kinds of thoughts, this kind of situation might indicate that dreams expressed positive spontaneous thoughts indirectly. Here, we also hypothesized that presleep focusing on positive spontaneous thoughts enhanced the possibility of dreaming of thoughts that were related to the positive spontaneous thoughts but not the positive spontaneous thoughts themselves.

## Materials and methods

### Participants

At first, a total of 100 participants were selected to participate in this study. These participants were recruited by online advertisement toward students of the local university. If they met the following criteria, they would be accepted for the study: high dream recall frequency (more than two or three dreams per week); easy to fall asleep (within 30 min); stable sleep period (at least 6 h each night); and no neurological or psychiatric history. Based on the time of signing up, participants were equally divided into two groups. All subjects gave written informed consent before the start of the study, and the Research Ethics Committee of the local university approved the study [SCNU-PSY-2021-118].

Among the 100 participants, five of them failed to recall their dreams, two of them failed to finish the night task at night, and three of them did not finish the night task within enough time (5 minutes), so these participants were not included in the research. Finally, the number of the participants was 90: the expression condition (*N* = 45; 4 males, 41 females; mean [SD, range] age 20.53 [2.34, 18–27] years), and a control group (*N* = 45; 6 males, 39 females; mean [SD, range] age 20.31 [2.49, 18–29] years).

### Night task

The “Night Task” asked participants to identify and describe in detail their most pleasant and positive spontaneous thoughts. The positive spontaneous thought was that a thought of image that participants did not intend to think about, but pops into their head involuntarily. In addition, the emotional valence of the spontaneous thought should be positive.

Participants rated the degree of pleasantness caused by the spontaneous thoughts with a five-point Likert-scale and reported how many days in the past week they had the spontaneous thoughts.

Participants in the expression condition engaged in a 5-min stream of consciousness writing task in which they tried to think about the positive spontaneous thought and wrote down all thoughts in the process. Participants in the control condition engaged in a similar task to the expression condition, except they were not told to focus on the positive spontaneous thought. The specific instructions of the two conditions were adapted from Wang et al. (2022).

### Morning task

Similar to Wang et al. (2022), the “Morning Task” asked participants to record their dreams immediately after they waked up. Moreover, with five-point Likert-scales they rated the degree of the pleasantness of the dream, the degree of the emotional intensity of the dream, and the degree of the correlation between the dream and the positive spontaneous thought.

### Procedure

At first, participants received an eligibility questionnaire and an information sheet (detail see “Participants”). If they met the criteria they would get specific instructions for the experiment *via* email. Participants were quasi-randomly assigned to one of two conditions: expression or control. By a telephone or a computer, participants finished the experiment in an online web. Before the formal experiment, participants entered a practice exercise for the stream of consciousness task. The expression condition focused on thinking of a white bear within a period of 5-min. By contrast, firstly the control condition thought about a white bear and then they thought about anything they wanted within a period of 5-min. At the same time, both of the conditions finished a stream of consciousness task, which required participants to write down anything they thought during the task. In addition, each time they thought about the white bear they should make check marks. After the exercise, participants got two online instructions: one labeled “Night Task” and the other labeled “Morning Task.” The specific content of the “Night Task” was shown in “Night task.” Participants completed the “Morning Task” immediately after waking. The content of the task was shown in “Morning task.” Then two judges who were blind to participants’ group and status rated similar elements (characters, objects, locations, and activities) between spontaneous thoughts and dreams. In addition, the judges also rated if there were similar elements (characters, objects, locations, and activities) between dreams and thoughts related to spontaneous thoughts but not spontaneous thoughts themselves (detail see “Content analysis for the correlation between a spontaneous thought and a dream”). The two judges were psychological postgraduates and both of them had experiences for the content analysis method. For the two kinds of rating, the Cronbach’s consistency coefficient was 0.82 and 0.78 separately. All inconsistent rating results were discussed later until reaching an agreement.

### Content analysis for the correlation between a spontaneous thought and a dream

Similar to Wang et al. (2022), the self-rating instructed participants to rate the degree of the correlation between the dream and the positive spontaneous thought.

The external-rating instructed external judges to rate similar characters, objects, locations, and activities between positive spontaneous thoughts and dreams. The same ‘I’ was not viewed as similar characters between the thoughts and dreams. The operational definitions for the external-rating were in the Appendix 1. In the rating process, external judges identified potential similar elements in a literal way. That meant only the category of the positive spontaneous thought that had been expressed clearly would be used for further rating. For example, consider the following positive spontaneous thought: my girlfriend. In this case, there was a character category, without any other kinds of categories (objects, locations, and activities). So the external-rating method identified if dream content contained characters that were similar to the girlfriend.

In addition, as stated in the “Introduction,” in this study we also rated correlations between dreams and thoughts that were related to positive spontaneous thoughts but not the positive spontaneous thoughts themselves. This kind of thought was represented by words around positive spontaneous thoughts that were reported during the stream of consciousness task. Specifically, during the stream of consciousness task participants reported word strings. As the positive spontaneous thought was marked with check marks in the word strings, in the word strings, words around the check marks within one word were seen as thoughts related to positive spontaneous thoughts but not positive spontaneous thoughts themselves. For example, the positive spontaneous thought was ‘white bear’, and the word string reported during the stream of consciousness task was the following: ‘white bear’, ‘animal’, ‘white color’, ‘snow’, ‘white bear’, ‘meat’, ‘nature’. In the word string, words around the check marks within one word were the following: ‘animal’, ‘snow’, and ‘meat’. By contrast, the ‘white color’ and the ‘nature’ were words that were not around the check marks within one word. So in this case the ‘animal’, the ‘snow’, and the ‘meat’ were thoughts related to positive spontaneous thoughts but not positive spontaneous thoughts themselves. The external-judges also rated if there were similar elements (characters, objects, locations, and activities) between dreams and thoughts related to positive spontaneous thoughts but not positive spontaneous thoughts themselves.

### Statistical analysis

For data of the self-rating, the Wilcoxon test was used to make group comparisons between the expression condition and the control condition. Effect sizes were calculated as *z*/√(*n*) where *z* is the *z*-statistic and *n* the number of observations (i.e., 45 per subgroup).

Similar to Wang et al. (2022), for data of the external-rating, a score of 0 for no presence of similar elements or 1 for the presence of similar elements was used. If there were more than one type of similar element between a major concern and a dream, the degree of the correlation between the major concern and the dream was still scored as 1. The chi-square test was used for group comparisons between the expression condition and the control condition.

According to Freud (1900), different dreams of a single night may refer to a similar topic. So in this study, each participant’s dreams were analyzed based on a single night, regardless of the number of the night’s dreams. For example, one participant reported two dreams of a single night, then the two dreams were viewed as one whole dream text for the statistical analysis. As a result, each participant provided one rating score for both the self-rating and the external rating.

## Results

For the expression condition, the dream length was 151.13 (SD = 165.99) words. For the control condition, the dream length was 143.71 (SD = 150.24) words.

### Self-rating

The descriptive data of the self-rating are reported in Table 1. Wilcoxon tests indicated that the two groups did not differ on age, pleasantness of positive spontaneous thought, frequency of positive spontaneous thought, dream length, dream valence, and dream emotional intensity (all *p* > 0.20). Compared with the control condition, the expression condition reported a higher number of the positive spontaneous thought (*z* = 3.55, *p* < 0.001, *effect size* = 0.37). So our manipulation was effective.

**Table 1:** Mean (SD) for descriptive data of different variables of the self-rating.

Category	Expression condition	Control condition
	Mean (SD)	Mean (SD)
Pleasantness of positive spontaneous thoughts^a^	4.11 (0.78)	4.11 (0.88)
Frequency of positive spontaneous thoughts	3.78 (1.80)	3.53 (1.69)
Number of presleep positive spontaneous thoughts	6.73 (4.78)	3.47 (2.06)
Dream valence^b^	3.07 (1.00)	3.02 (1.01)
Dream emotional intensity^c^	3.40 (0.69)	3.29 (0.69)

a 1 (not at all pleasantness) to 5 (extremely pleasantness).

b 1 (very unpleasantness) to 5 (very pleasantness).

c 1 (not at all intense) to 5 (extremely intense).

The self-rating method found a significant difference in the correlation between the dream and the positive spontaneous thought among the two conditions (*z* = 0.90, *p* = 0.37).

### External-rating

The descriptive data of the external-rating are reported in Table 2. For the expression condition, 40% of dreams were rated as correlating with positive spontaneous thoughts, and 66.7% of dreams were rated as correlating with thoughts related to positive spontaneous thoughts but not positive spontaneous thoughts themselves. For the control condition, 20% of dreams were rated as correlating with positive spontaneous thoughts, and 40% of dreams were rated as correlating with thoughts related to positive spontaneous thoughts but not positive spontaneous thoughts themselves.

**Table 2:** The observation (percentage) for descriptive data of the external-rating.

Category	Expression condition^a^	Control condition^a^
	Observation (frequency)	Observation (frequency)
Correlation between dreams and positive spontaneous thoughts	18 (40%)	9 (20%)
Correlation between dreams and thoughts related to positive spontaneous thoughts^b^	30 (66.7%)	18 (40%)

a The total number of observation in the expression condition was 45, and the total number of observation in the control condition was 45.

b Thoughts related to positive spontaneous thoughts but not positive spontaneous thoughts themselves, for detail see “Content analysis for the correlation between a spontaneous thought and a dream.”

The expression reported a higher proportion of the correlation between dreams and positive spontaneous thoughts, than the control condition (*χ^2^* = 4.29, *p* = 0.038, *phi* = 0.22). In addition, the expression reported a higher proportion of the correlation between dreams and thoughts related to positive spontaneous thoughts but not positive spontaneous thoughts themselves, than the control condition (*χ^2^* = 6.43, *p* = 0.011, *phi* = 0.27).

## Discussion

In this study, we found that presleep focusing on positive spontaneous thoughts enhanced the possibility of dreaming of the thoughts. This result was found by the external-rating method rather than the self-rating method. A similar situation happened in Wang et al. (2022) in which only the external-rating method found that presleep focusing on intrusive thoughts enhanced the possibility of dreaming of the thoughts. As stated in the “Introduction,” the external-rating method may be more suitable for group comparisons than the self-rating method. As there are dream metaphors that represent waking-life experiences indirectly (see Malinowski and Horton, 2015), the self-rating method may lead to potential errors caused by individual differences in rating dream metaphors, which, in turn, may affect results for group comparisons. By contrast, as the external-rating method used a similar standard to rate dream metaphors, the errors caused by the self-rating method could be reduced, and thus the external-rating method was more suitable for group comparisons.

According to the current-concern theory, a current concern increases the individual’s sensitivity toward cues related to the concern, which, in turn, enhances the possibility for the concern being processed (e.g., see Klinger, 2013). Recent evidence that may support this theory is findings concerning the topic of the influence of the pandemic (COVID-19) on dreams. It has been shown that COVID-19 affects dream characteristics (for a review, see Gorgoni et al., 2022), such as dream recall frequency (e.g., see Scarpelli et al., 2022b), and the continuity between waking concerns and dream content (e.g., see Wang et al., 2021). During waking time, COVID-19 may be a current concern, which, in turn, affects dream characteristics. The spontaneous thought is argued to be related to a current concern (see Klinger, 2013). Presleep focusing on current concerns enhanced the possibility of dreaming of them (e.g., see Nikles et al., 1998), so the instruction to focus on a positive spontaneous thought led to more dreams concerning the topic of the positive spontaneous thought. To some degree, our result supported the current concern theory.

In addition, the continuity hypothesis suggests that dreaming is in continuous with waking life (e.g., Hall and Nordby, 1972; Domhoff, 2003, 2017; Schredl, 2003, 2012). The evidence that supports the continuity hypothesis comes from a basic hypothesis that the frequency of a dream element is associated with the intensity of a waking-life experience related to the dream element (e.g., see Domhoff, 2017). There are two versions of the continuity hypothesis of dreaming. On the one hand, one version of the continuity hypothesis is related to a long-term perspective, which describes that dreams express personal long-term concerns and conceptions (e.g., see Domhoff, 2017). On the other hand, the other version of the continuity hypothesis suggests that the continuous association between waking and dreaming is not only referred to a long-term period of time but also a single day. From the long-term perspective, this version of the continuity hypothesis indicates that the intensity of personal waking-life experiences may be associated with the frequency of dream element related to the waking-life experiences (e.g., see Schredl, 2012). From the perspective of a single day, our results supported the continuity hypothesis of dreaming (e.g., Hall and Nordby, 1972; Schredl, 2012).

Besides, we found that presleep focusing on positive spontaneous thoughts enhanced the possibility of dreaming of thoughts that were related to positive spontaneous thoughts but not positive spontaneous thoughts themselves. The thoughts that were related to positive spontaneous thoughts but not positive spontaneous thoughts themselves were measured by thoughts that happened next to spontaneous thoughts in the stream of conscious task (detail see “Procedure”). Dreaming is involved with autobiographical memory which is the memory that is related to oneself (e.g., see Malinowski and Horton, 2015). A characteristic of the long-term autobiographical memory is the coherence that a memory representation is coherent with other memory representations of the self (true to the self; e.g., see Conway and Loveday, 2015). From the perspective of a personal long-term memory network, there is some subjective meaning of coherence between a positive spontaneous thought and a thought that is recalled next to the positive spontaneous thought. In other words, the positive spontaneous thought and the thought recalled next to the positive spontaneous thought may share some subjective similarities. So the appearance of a thought next to a positive spontaneous thought in dreams may indicate that dreams express the positive spontaneous thought indirectly, or metaphorically. Thus, our result suggested that presleep focusing on positive spontaneous thoughts enhanced the possibility of dreaming of the thoughts metaphorically. This result supported the current concern theory which suggests that one’s current goal enhances responses to cues that are related to the concern implicitly, since spontaneous thoughts are argued to be related to current concerns (Klinger, 2013). In addition, as stated above, from the perspective of a single day, the continuity hypothesis indicates that the intensity of personal waking-life experiences may be associated with the frequency of dream element related to the waking-life experiences (e.g., Hall and Nordby, 1972; Schredl, 2012). Here, our finding also supported this version of the continuity hypothesis.

We note several limitations of the present research. First, we focused on positive spontaneous thoughts and thus it is unclear whether similar processes may occur for thoughts that are not spontaneous. Future study should investigate if presleep focusing on non-spontaneous thoughts (such as important waking-life experiences) can affect the possibility of dreaming of the thoughts. Second, the dreams were collected at home. So far, some research has found that there are some correlations between specific EEG patterns and dream characteristics, such as the incorporation of waking experiences into dreams (e.g., Eichenlaub et al., 2018; for a review, see Scarpelli et al., 2022a). This kind of laboratory research can help to provide more insights for potential neurobiological mechanism of dreaming. Future research could be conducted in a sleep laboratory. In addition, participants adopted here were mainly female. Future research could use more male participants for repetitive purpose. Moreover, in this study, the self-rating method was to instruct participants to give a single score to represent the correlation between a positive spontaneous thought and a dream. Future study could use other kinds of method for the self-rating, such as the way of the external-rating method adopted here, which instructed participants to identify similar elements between a positive spontaneous thought and a dream (e.g., see Wang et al., 2020).

In conclusion, the current study found that presleep focusing on positive spontaneous thoughts enhanced the possibility of dreaming of the thoughts. In addition, presleep focusing on positive spontaneous thoughts enhanced the possibility of dreaming of thoughts that were related to the positive spontaneous thoughts but not the positive spontaneous thoughts themselves. These results supported the current concern theory (e.g., Klinger, 2013). In addition, as one version of the continuity hypothesis describes the influence of daily activities on dreams (e.g., Hall and Nordby, 1972; Schredl, 2012), the above results also supported this version of the continuity hypothesis.

## Data availability statement

The original contributions presented in the study are included in the article/Supplementary material, further inquiries can be directed to the corresponding author.

## Ethics statement

The studies involving human participants were reviewed and approved by the Ethics Committee of the South China Normal University. The patients/participants provided their written informed consent to participate in this study.

## Author contributions

JW: design and wrote the manuscript. JW, XF, and RL: data collection and data analysis. BS and HS: edit the manuscript. All authors contributed to the article and approved the submitted version.

## Funding

Supported by the Humanities and Social Sciences of Guangzhou University of Chinese Medicine (2020SKXK17).

## Conflict of interest

The authors declare that the research was conducted in the absence of any commercial or financial relationships that could be construed as a potential conflict of interest.

## Publisher’s note

All claims expressed in this article are solely those of the authors and do not necessarily represent those of their affiliated organizations, or those of the publisher, the editors and the reviewers. Any product that may be evaluated in this article, or claim that may be made by its manufacturer, is not guaranteed or endorsed by the publisher.
