# Supplementary material for: Presleep focusing on positive spontaneous thoughts enhanced the possibility of dreaming of them
Source: Front Psychol. 2022 Dec 14;13:1042857. doi: 10.3389/fpsyg.2022.1042857 (PMC9794732; doi:10.3389/fpsyg.2022.1042857)
Supplement: Supplementary file 1 [file Data_Sheet_1.docx]

Appendix 1

1.Characters: consist of people, animals, or mythical figures.

1.1.Relatives: a person who has a relative relationship with the dreamer (note that in this subcategory, only characters that belong to a same subcategory (1.1.1. to 1.1.4.) are rated as similarity).

1.1.1.Previous generation: a person who is the previous relative generation of the dreamer, such as father, mother, stepmother, foster father, grandmother, grandfather, uncle, and aunt etc..

1.1.2.Peer relatives: a person who is the peer relative of the dreamer, such as brother, sister, cousin, half brother, and half sister etc..

1.1.3.Next generation, a person who is the peer relative of the dreamer, such as child, son, daughter, nephew, and niece etc..

1.1.4.marriage-like relatives, a person who may have a marital-like relationship with the dreamer, such as girlfriend, boyfriend, husband, wife, ex-grilfriend, ex-boyfriend, ex-husband, and ex-wife etc..

1.2.Friends: a person who is a friend of the dreamer, such as friend, close friend etc..

1.3.Classmates: a person who is a classmate of the dreamer, such as classmate etc..

1.4.Roommates: a person who is a roommate of the dreamer, such as roommate etc..

1.5.Vocation: any character whose occupation is designated, such as teacher, doctor etc. (Note that in this class, only characters that belong to a same vocation are rated as similarity).

1.6. Prominent: any character who is well known by her or his general reputation but who is not known personally by the dreamer. In addition, fictional, dramatic, imaginary, and supernatural figures are also coded under this heading as they are usually familiar because of their reputation.

1.7.Animals.

1.8.Others: other kind of character which is not included in the above categories. This category requires that the character in a dream report is described clearly the same with the character in a waking-life experience. For example, dream: I went to a library with A, and waking-life experience: I talked with A. In this case, the person A is counted in this category.

2.Objects: consist of objects.

2.1.Household: all objects frequently encountered in a household setting. Such as furniture, table, chair, and bed; appliances such as stove, refrigerator, and vacuum cleaner; furnishings such as rug, drapes, and lamp; Supplies such as sheet, light bulb, and soap. Tableware, kitchenware, and bathware etc..

2.2.Food and drinks: food, drinks, and also including general terms, such as groceries, things to eat, and a meal etc..

2.3.Implements: a tool that is needed in some specific activities (note that in this subcategory, only objects that belong to a same subcategory (2.3.1. to 2.3.4.) are rated as similarity).

2.3.1.Weapon tools: objects that are weapons, such as gun and sword etc..

2.3.2.Sporting objects: objects that are used in sporting, such as baseball bat and ball.

2.3.3.Playing objects: objects that are used in playing games, such as cards, checkers, dice, and toys etc..

2.3.4.Artistic objects: objects that are used in artistic activities, such as piano and drawing board etc..

2.4.Vocational: objects that are related to a work. For example, for a student, typical objects may be book, research, thesis etc. (Note that in this class, only objects that belong to a same vocational are rated as similarity).

2.5.Travel: all forms of conveyance, such as car, subway, train, airplane, bicycle. In addition, objects associated with above travel objects are also included, such as bus depot, train station, airport, license plate, passenger ticket etc..

2.6.Clothing: covered within this class are clothing and parts of clothing. Included are outer garments, underwear, headgear, and footwear, as well as such items as pocket, collar, and button etc..

2.7.Media: composed of all forms of visual, auditory, and written media and the means for transmitting them, such as TV, film, newspaper etc..

2.8.Money: this class incorporates money and objects closely associated with money, such as money and check etc..

2.9.Others: other kind of object which is not included in the above categories. This category requires that the object in a dream report is described clearly the same with the object in a waking-life experience. For example, dream: I saw some flowers, and waking-life experience: I saw flowers. In this case, the flowers is counted in this category.

3.Locations: consists of locations.

3.1.Residential: buildings and units of buildings (rooms) that are used for residential purposes, such as house etc..

3.2.Vocational: buildings and rooms in buildings devoted to work or occupation, such as store, factory, office, classroom, hospital, jail, and church etc..

3.3.Entertainment: buildings and rooms that are used for recreation, entertainment, or other pleasurable activities, such as theater, museum, art gallery etc..

3.4.Sporting: buildings and rooms that are used for sports, or exercise, such as gymnasium, swimming poor etc..

3.5.Road: all types of roadways by which a person can go from one place to another, such as street, road and bridge etc..

3.6.Others: other kind of location which is not included in the above categories. This category requires that the location in a dream report is described clearly the same with the location in a waking-life experience. For example, dream: I was at A, and waking-life experience: I went to A. In this case, the A is counted in this category.

4.Activities: what characters do is presented.

4.1.Vocational: typical activities that are related to a work. For example, for a student, typical activities may be studying, looking at books, having exam etc. For a teacher, typical activities may be teaching, assigning homework etc. For a doctor, typical activities may be having an operation, seeing patient etc. (Note that in this class, only activities that belong to a same vocational are rated as similarity).

4.2.Art: activities that are related to the art, such as dancing, singing, and drawing etc..

4.3.Entertainment: activities that are related to the entertainment, such as going shopping, traveling, and playing etc..

4.4.Household: activities that are related to the household, such as cleaning and tidying up etc..

4.5.Sporting: activities that are related to the sporting, such as running, climbing, playing balls and swimming etc..

4.6.Social interaction: activities that are related to the social interaction (note that in this subcategory, only social interaction that belong to a same subcategory (4.6.1. to 4.6.2.) are rated as similarity).

4.6.1.Aggression: social negative interaction that can be verbal aggression, such as cursing etc., or physical aggression, such as attacking etc..

4.6.2.Friendliness: social positive interaction that can be verbal friendliness, such as encouraging etc., or physical friendliness, such as helping etc..

4.7.Self reflection: this subclass includes activities that are related to the self reflection, such as recalling the past, planning the future, and introspection etc..

4.8.Others: other kind of activity which is not included in the above categories. This category requires that the activity in a dream report is described clearly the same with the activity in a waking-life experience. For example, dream: I was bathing, and waking-life experience: I bathe for a long time. In this case, the bathing is counted in this category.
